# Draft Genome Sequence of *Agrobacterium* sp. Strain UHFBA-218, Isolated from Rhizosphere Soil of Crown Gall-Infected Cherry Rootstock Colt

**DOI:** 10.1128/genomeA.00302-13

**Published:** 2013-05-30

**Authors:** Ankita Dua, Naseer Sangwan, Jasvinder Kaur, Anjali Saxena, Puneet Kohli, A. K. Gupta, Rup Lal

**Affiliations:** Department of Zoology, University of Delhi, Delhi, Indiaa; Department of Mycology and Plant Pathology, Dr. Y. S. Parmar University of Horticulture and Forestry, Nauni, Solan, Himachal Pradesh, Indiab

## Abstract

We report here the draft genome sequence of the alphaproteobacterium *Agrobacterium* sp. strain UHFBA-218, which was isolated from rhizosphere soil of crown gall-infected cherry rootstock Colt. The draft genome of strain UHFBA-218 consists of 112 contigs (5,425,303 bp) and 5,063 coding sequences with a G+C content of 59.8%.

## GENOME ANNOUNCEMENT

Species of the genus *Agrobacterium* have been implicated either in the formation of tumors (galls) or for the production of agrocin-like antibiotics that suppress the tumor caused by them (1). We isolated *Agrobacterium* strain UHFBA-218 from rhizosphere soil of crown gall-infected cherry rootstock Colt (April 2010). This strain did not show any gall development on an artificially inoculated stem of a potted tomato plant when tested for pathogenicity (2) and was designated a nonpathogenic isolate belonging to the biovar I division of *Agrobacterium* (genome species G6). Strain UHFBA-218 is available from Microbial Culture Collection, Pune, India (MCC2101), and the current draft genome is listed in Genomes OnLine Database (GOLD) as card Gi23696.

The genome of *Agrobacterium* strain UHFBA-218 was sequenced using Illumina (2-kb paired-end library; 6,733,683 reads) and 454 GS FLX titanium (74,904 reads) platforms. The data were assembled using the Velvet 1.2.03 assembler (3) set at a k-mer length of 55. The assembly generated 112 contigs (*N*_50_ contigs, 100.18 kb) comprising of 5,425,303 bp. The final (validated pair-end criterion) assembly had 85.67-fold coverage with the largest contig (GenBank accession no. APCC01000003) of 246,613 bp in size and an average G+C content of 59.8%.

The draft genome was annotated using RAST version 4.0 (4), NCBI Prokaryotic Genomes Automatic Annotation Pipeline (PGAAP) (http://www.ncbi.nlm.nih.gov/genomes/static/Pipeline.html). A total of 5,063 protein-coding sequences and 1,412 hypothetical proteins were predicted on annotation. A single copy of each of the 5S, 16S, and 23S RNA and 56 tRNA genes was predicted using RNAmmer (5) and tRNAscan-SE (6), respectively. The contig 21 (GenBank accession no. APCC01000021) contains an operon which is >97% similar to the characterized RNA operon reported in *A. tumefaciens* MAFF301001 (GenBank Accession no. AB102734.2) and codes for 16S rRNA, tRNA-Ile-GAT, tRNA-Ala-TGC, 23 S rRNA, 5S rRNA, and tRNA-Met-CAT. The draft genome was found to be closely related to *A. tumefaciens* C58 (88% nucleotide identity as determined by using ANI perl script [7]). Contig 26 (GenBank accession no. APCC01000026) of the final (validated) assembly was found to contain prophage genomic regions (15.3 kb) as predicted using PHAST (8). S1-PFGE analysis (9) of strain UHFBA-218 indicated a possible tripartite genomic makeup, with a primary circular chromosome, a secondary linear chromosome, and the presence of a megaplasmid. Based on BLASTn analysis, a 149-kb cluster of contigs (*n* = 12) represented a megaplasmid that showed 99% homology (51% query coverage) to the tumor-inducing plasmid pTiC58. However, no virulence genes have been found on the putative megaplasmid, which makes it an interesting prospect for future research.

A detailed comparative genomic analysis of strain UHFBA-218 and its phylogenetic neighbors (10) that belong to biovar I is underway. Such analysis is expected to provide further insights into the evolution of the genomic organization pertaining to this biovar designation.

### Nucleotide sequence accession numbers.

This Whole-Genome Shotgun project has been deposited at DDBJ/EMBL/GenBank under the accession number APCC00000000. The version described in this paper is the first version, APCC01000000.
